# Surgical Anatomy

**Published:** 1851-11

**Authors:** 


### REVIEWS.

---

Art. IV.—*Surgical Anatomy.* By Joseph Maclise, Surgeon. With colored plates. Part iv. Philadelphia: Blanchard & Lea. 1851.

We have spoken of the preceding numbers of this important work in terms of warm commendation, and have endeavored to impress upon the minds of medical practitioners, the strong claims the labors of Mr. Maclise have upon them. Within the range of our observation, there is not a work on any of the specialties of anatomy superior to the surgical anatomy of Maclise. The hand of a master is displayed alike in the general result, and in the most minute details of these plates. As a work for consultation in many of the sudden and perplexing emergencies that spring upon the physician, this has no rival. These views of the utility and importance of Maclise's plates have been impressed upon us from a careful examination of parts 1, 2 and 3 of the work, but the plates of part 4 are, if possible, more important to the general practitioner than those of the preceding numbers. The following list of the subjects illustrated in the number before us, shows the character of the instruction to be derived from the plates of Maclise.

Plate 47.—"The surgical dissection of the principal blood-vessels and nerves of the Iliac and Femoral Regions." This admirable plate is accompanied with a clear and forcible commentary by the author, and the remarks are all that can be desired in the way of useful instruction.

Plates 48 and 49.—The relative anatomy of the male pelvic organs, with a commentary.

Plates 50 and 51.—"The surgical dissection of the superficial structures of the male perinæum."

Plates 52 and 53.—"The surgical dissection of the deep structures of the male perinæum. The lateral operation of lithotomy."

These are excellent plates, and the accompanying commentary is filled with valuable truths and useful instruction in connection with lateral lithotomy.

Plates 54, 55 and 56.—"The surgical dissection of the male bladder and urethra—lateral and bilateral lithotomy compared." The relations of catheterism are admirably portrayed in these plates.

Plates 57 and 58.—"Congenital and pathological deformities of the prepuce and urethra—stricture and mechanical obstructions of the urethra."

The various figures of these plates are as useful to the general practitioner, as any of the preceding ones are to the surgeon. In plate 57, there are fifteen forms of preputial and urethral congenital or pathological deformities, and some of them require all the knowledge that can be brought to bear upon them, for success in remedial efforts. Congenital and acquired phymosis, hypospadias, epispadias, and various urethral deformities are admirably illustrated in the fifteen figures that occupy plate 57. The surgical advice given by Mr. Maclise, for the management of these deformities is sound, and worthy of confidence. In the commentary on figure fifteen, Mr. Maclise seems to adopt Mr. Syme's commendation of the perineal section for stricture, and we have no doubt of the value of that contribution to the surgery of some forms of stricture. We speak of "some forms," because we are satisfied that there are cases of stricture that are much more benefitted by constitutional treatment, than by any local applications. There is no department of surgery that needs more thorough steady and careful observation than that which is devoted to the treatment of strictures of the urethra. When Mr. Abernethey reviewed the various contrivances for the relief of urethral stricture, he thought the climax of absurdity had been reached in the proposition to convert the urethra into a soap factory, that being the meaning of the use of caustic in its application to the stricture. Yet, that absurdity has its advocates in some of the highest quarters of surgery.

We are convinced that there is entirely too much instrumental work in the management of strictures of the urethra. They are the opprobria of surgery, not because the science is incapable of managing them, but because its mechanical powers are attempted, where its philosophy alone is needed, and where it is fully equal to the desired success. We admire the zeal and earnest efforts of Mr. Syme in favor of what he considers a great improvement in the management of stricture of the urethra, and we doubt not that his suggestion in favor of his peculiar operation, is one advance upon other forms of instrumental assistance. But we are satisfied that too much is hoped for from instruments, and our limited observation has taught us that in the great majority of cases, constitutional treatment is worth all the other methods of managing stricture of the urethra that have ever been devised. Judicious medication, proper diet, perseverance and patience are the sure methods for permanent cure of stricture.

Plate 58.—The figures in this plate, eleven in number, are illustrative of singular forms of stricture, and of calculi lodged in the bladder. These two plates will amply repay a long, and rigid study.

Plates 59 and 60.—"The various forms and positions of strictures and other obstructions of the urethra—false passages—enlargements and deformities of the prostate."

The delineations in these plates are of great value to the general practitioner—they constitute a chart of the highest importance to those who have much to do with the intricate and obscure navigation of the parts involved in the deformities depicted. In order to show the character of this part of the work, we shall enter into some details.

Figs. 1 and 2, plate 59, present seven forms of organic stricture occurring in different parts of the urethra. They constitute a study of great importance, and will, we hope, attract the attention of physicians.

Fig. 3, represents three separate strictures in the urethra, anterior to the bulb.

Fig. 4. "In this the canal is constricted at a point midway between the bulb and glans. A false passage has been made under the urethra by an instrument which passed out of the canal, anterior to the stricture, and reëntered the canal anterior to the bulb.

Fig. 5. The stricture appears midway between the bulb and glans, the area of the passage through the stricture being sufficient only to admit a bristle to pass.

Fig. 6. Two instruments have made false passages beneath the mucous membrane, in a case where no stricture at all existed.

Fig. 7. A bougie perforates the urethra anterior to the stricture, situated an inch behind the glans, and after traversing the substance of the right corpus cavernosum for its whole length, reënters the neck of the bladder through the body of the prostate.

Fig. 8. A bougie appears tearing and passing beneath the lining membrane of the prostatic urethra. The origin of the false passage is in general anterior to the stricture. It may, however, occur at any part of the canal in which no stricture exists, if the hand that impels the instrument be not guided by a true knowledge of the form of the urethra; and perhaps the accident happening from this cause is the more general rule of the two.

Fig. 9. Two strictures are represented here, the one close to the bulb, the other an inch anterior to this part. In the prostate are seen irregularly shaped abscess pits, communicating with each other, and projecting upwards the floor of this body to such a degree, that the prostatic canal appears nearly obliterated.

Fig. 10. Two bougies are seen to enter the upper neck of the urethra anterior to the prostate. This accident happens when the handle of a rigid instrument is depressed too soon, with the object of raising its point over the enlarged third lobe of the prostate.

Fig. 11. Two instruments appear transfixing the prostate, of which body the three lobes are much enlarged; the instrument perforates the third lobe, while the instrument penetrates the right and the third lobe. This accident occurs when instruments not possessing the proper prostatic bend are forcibly pushed forwards against the resistance at the neck of the bladder.

Fig. 12. In this case an instrument, after passing beneath part of the lining membrane anterior to the bulb, penetrates the right lobe of the prostate; a second instrument penetrates the left lobe; a third smaller instrument is seen to pass out of the urethra anterior to the prostate, and after transfixing the right vesicula seminalis external to the neck of the bladder, enters this viscus at a point behind the prostate. The resistance which the two longer instruments met with in penetrating the prostate, made it seem, perhaps, that a tight stricture existed in this situation, to match which the smaller instrument referred to was afterwards passed in the course marked out.

Plate 60.—Figs. 1 to 5, represent a series of prostates, in which the third lobe gradually increases in size. In figure 1, which shows the healthy state of the neck of the bladder, unmarked by the permanent lines which are said to bound the space named "trigone vesical," or by those which indicate the position of the "muscles of the ureters," the third lobe does not exist. In figure 2, it appears as uvula vesicæ. In figure 3, this part is increased, and under the name now of third lobe is seen to contract and tend upwards the prostatic canal. In figure 4, the effect which the growth of the lobe produces upon the form of the neck of the bladder becomes more marked, and the part presenting perforations, produced by instruments, indicates that by its shape, it became an obstacle to the egress of the urine as well as to the entrance of instruments. In figure 5, the three lobes are enlarged, but the third is most so, and while standing on a narrow pedicle attached to the floor of the prostate, completely blocks up the neck of the bladder.

Fig. 6. The prostatic canal is bent upwards by the enlarged third lobe to such a degree as to form a right angle with the membranous part of the canal. A bougie is seen to perforate the third lobe, and this is the most frequent mode in which, under such circumstances, and with instruments of the usual imperfect form, access may be gained to the bladder for the relief of the retention of urine.

Fig. 7. The three lobes of the prostate are equally enlarged; the prostatic canal is consequently much contracted and distorted, so that an instrument on being passed into the bladder has made a false passage through the third lobe. When a catheter is suspected to have entered the bladder by perforating the prostate, the instrument should be retained in the newly made passage, till such time as this has assumed the cylindrical form of the instrument. If this be done, the new passage will be the more likely to become permanent. It is ascertained that all false passages and fistula by which the urine escapes, become after a time lined with a membrane similar to that of the urethra.

Fig. 8. The three lobes of the prostate are irregularly enlarged; the third lobe projecting from below, distorts the prostate canal upwards and to the right side.

Fig. 9. The right lobe of the prostate appears hollowed out, so as to form the sack of an abscess which, by its projection behind, pressed upon the fore part of the rectum, and by its projection in front contracted the area of the prostatic canal, and thereby caused an obstruction in this part. Not unfrequently when a catheter is passed along the urethra, for the relief of a retention of urine, caused by the swell of an abscess in this situation, the sac becomes penetrated by the instrument, and, instead of urine, pus flows. The sac of a prostatic abscess frequently opens of its own accord into the neighboring part of the urethra, and when this occurs, it becomes necessary to retain a catheter in the neck of the bladder to prevent the urine entering the sac.

Fig. 10. The prostate presents four lobes of equal size, and all projecting largely around the neck of the bladder; the prostatic canal is almost completely obstructed, and an instrument has made a false passage through one of the lobes.

Fig. 11. The third lobe of the prostate is viewed in section, and shows the track of the false passage made by a catheter, through it from its apex to its base; the proper canal is bent upwards from its usual position, which is that at present marked by the instrument in the false passage.

Fig. 12. The prostatic lobes are uniformly enlarged, and cause the corresponding part of the urethra to be uniformly contracted, so as closely to embrace the catheter, occupying it, and to affix considerable resistance to the passage of the instrument.

Fig. 13. The prostate is considerably enlarged anteriorly, in consequence of which, the prostatic canal appears more horizontal even than natural. The catheter occupying the canal lies nearly straight. The lower neck of the prostate is much diminished in thickness. A nipple shaped projection is attached by a pedicle to the back of the upper part of the prostate, and acts like a stopper to the neck of the bladder. The body being moveable, it will be perceived how, while the bladder is distended with urine, the pressure from above may blow up the neck of the organ with this part, and thus cause complete retention, which, on the introduction of a catheter, becomes readily relieved by the instrument pushing the obstructing body aside.

We have entered into these details from a conviction of their important bearing upon practical medicine, and a knowledge of the many grievous mistakes and disasters that are consequent upon ignorance of the matters illustrated by Mr. Maclise, in the 59th and 60th plates of his admirable work. We have seen some of the evils of which this author speaks, and we have heard of many instances where the mischief was irreparable. The details we have given of the figures in these two plates are taken from Mr. Maclise's commentary, and are altered only to suit a publication without the plates. We indulge the hope that this plan will induce the practitioner to procure the work, and give his strict attention to the study of this series of instructive plates of surgical anatomy. The most complete knowledge that can be obtained of the deformities of the urethral canal, is not more than sufficient to qualify the practitioner of medicine for the performance of his duties to such of his patients as may be the victims of this class of disorders. It is the rare merit of Maclise that he has made the portion of his surgical anatomy, which we have minutely detailed, a series of clinical surgery beyond any that may be acquired in the curriculum of any school. We have pointed out where these treasures of knowledge may be sought, and the pleasure of pointing to this mine is greatly enhanced by the consciousness that those who truly seek will surely find.

Blundell, in his excellent lectures on obstetrics, impressively warns against the dangers of vaginal examinations, by assuring the obstetrician that he may inflict a wound as fatal as any received at Waterloo. If such a teacher as

Blundell could thus speak on such a subject, how trumpet-tongued should be the admonitory warnings on the dangerous explorations of the urethra! Who can survey the perils of such explorations, and know how ignorantly they are often attempted, without shuddering at the probable consequences!

Reader! if it is your province to manage diseases of the urinary organs, we pray your attention to the graphic instructions contained in the surgical anatomy of Maclise. They are lessons that can be acquired as well no where else. And while you look upon the disasters of urethral instrumentation, seek to find a philosophy that will eradicate these diseases without a resort to instruments of any kind. Shun them and save yourself and your patients from disaster.

We have been strongly impressed with the value of the instruction contained in the plates 59 and 60, and endeavored to point out that value by verbal descriptions. They are especially devoted to the urethra, and in a minor degree to the prostate gland. The succeeding and last plates of this number are devoted to "deformities of the prostate—distortions and obstructions of the prostatic urethra." This is a fine field, and it is very thoroughly worked by Maclise. But we cannot dwell upon the merits of the two plates devoted to the subjects we have named above.

The original intention was to complete this republication in four parts, but the publishers have been compelled to add another number, and the five numbers will be furnished at nine dollars, and they will be found to be about the best and cheapest outfit a physician can command for surgical practice. B.

